# Recommendations for prostate cancer diagnosis and treatment during COVID-19 outbreak were not followed in Brazil

**DOI:** 10.1590/S1677-5538.IBJU.2021.0673

**Published:** 2022-02-02

**Authors:** Fernando Korkes, Khalil Smaidi, Frederico Timoteo, Sidney Glina

**Affiliations:** 1 Faculdade de Medicina do ABC Santo André SP Brasil Disciplina de Urologia, Faculdade de Medicina do ABC - FMABC, Santo André, SP, Brasil;; 2 Hospital Israelita Albert Einstein São Paulo SP Brasil Serviço de Urologia, Hospital Israelita Albert Einstein, São Paulo, SP, Brasil

## COMMENT

In Brazil, the COVID-19 outbreak has spawned two intertwined massive waves of hospital admissions, which have exposed the long-acquainted public health care shortcomings in our country. Before the end of October 2021, Brazil had reached 21.680.488 confirmed severe acute respiratory syndrome cases due to coronavirus 2 (SARS-Cov-2), accounting for over 604.000 deaths, ranking Brazil the second in number of fatalities worldwide, only behind the United States with over 732.000 reported deaths. This significant overload in hospital admissions and intensive care unit need has resulted in incredible stress to hospitals across the country and compounded deep underlying problems in the Brazilian public health system. In this complex scenario, the health care structure should be prepared to respond to the SARS-Cov-2 increase of cases and other usual emergencies and other chronic conditions that require adequate diagnosis, workup, and treatment.

Several studies have demonstrated a decrease in non-COVID-related hospital admissions, including reductions in elective procedures ( 1 - 5 ). In Germany, non-COVID overall inpatient admissions decreased by 35% after the lockdown announcement, with even admissions for critical care conditions such as cancer treatment significantly reduced ( 4 ). A cross-sectional study in Brazil observed a significant reduction in hospital admissions related to cancer and cardiovascular, metabolic, and musculoskeletal diseases from January to June 2020 compared to the same period over the last three years. This study has observed a reduction as high as 35% in neoplasm inpatient admissions ( 6 ).

Non-melanoma skin cancer aside, Prostate Cancer (PCa) figures as the most prevalent neoplasm in men ( 7 , 8 ). In Brazil, according to GLOBOCAN, there were 97.278 PCa new cases in the year 2020, accounting for 16.4% of all neoplasm diagnoses in the same year ( 7 ). There are private and public health services in Brazil. The Public Health System (SUS) is responsible for the care of nearly 70% of all Brazilians. It is one of the largest Public Health Systems in the World. PCa burdens the health care system not only for its elevated prevalence but also because of disease characteristics. It demands populational screening with multiple clinic visits, prostate biopsies, imaging for staging, and finally, the treatment that comprehends surgery, radiotherapy, androgen deprivation therapy (ADT), and chemotherapy ( 9 ).

Some studies have demonstrated the impact of the COVID-19 pandemic on hospital admissions in chronic conditions and non-covid related emergencies ( 2 - 5 ). In Brazil, many health care institutions had their non-urgent procedures and elective surgeries suspended for months to concentrate economic and human resources to respond to the COVID-19 outbreak waves ( 10 ). With that, guidelines and suggestions have been provided to categorize urological diseases into risk groups and recommendations for follow-up during the COVID-19 outbreak in the management of numerous neoplasms ( 11 , 12 ).

**Figure 1:**
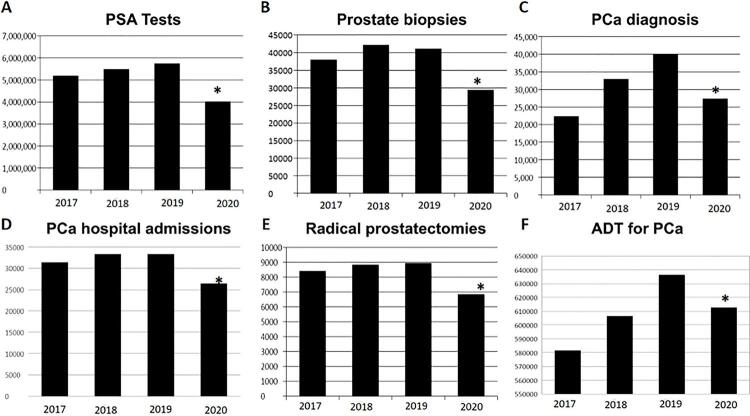
Number of patients in the Public Health System with a diagnosis of PCa in Brazil, from 2017 to 2020 and effects of the COVID-19 outbreak: A) Number of PSA tests (2017 to 2020); B) number of prostate biopsies (2017 to 2020); C) number of PCa diagnoses (2017 to 2020); D) number of hospital admissions with a diagnosis of PCa (2017 to 2020); E) number of radical prostatectomies performed (2017 to 2020); F) ADT for a diagnosis of PCa (2017 to 2020); *=p<0.05.

**Figure 2:**
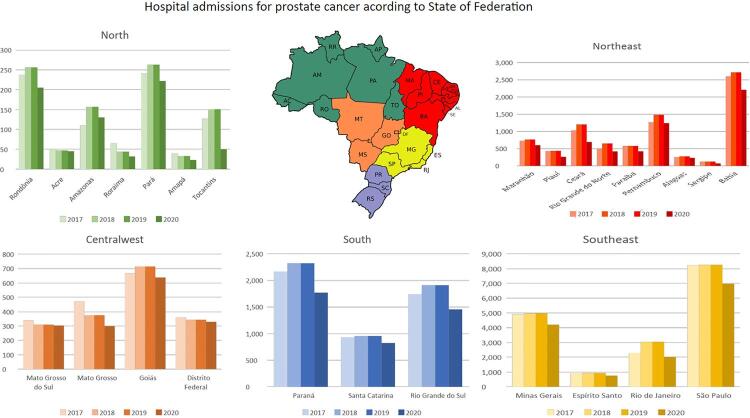
The number of hospital admissions for PCa increased during the last years until 2019, an average increment of 3.718% for PCa admissions was observed throughout Brazil from 2013 to 2019. But with the COVID-19 outbreak the hospital admissions for a diagnosis were also lower during 2020 than in 2019 in all twenty-seven states of Brazil.

**Figure 3:**
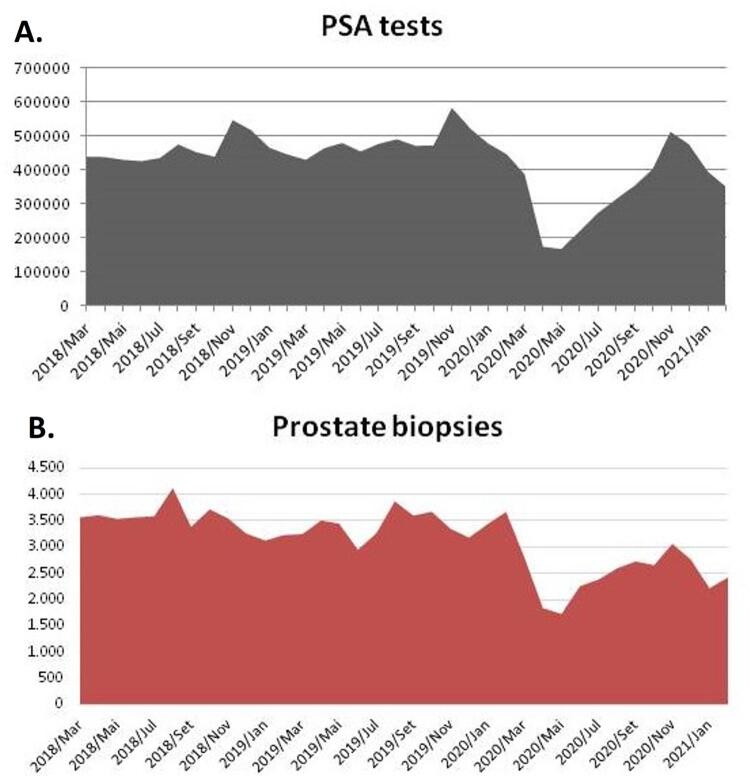
Number of PSA tests and Prostate Biopsies at the last three years, with a decrease during the COVID-19 outbreak.

In a cross-sectional evaluation obtained from the Brazilian Public Health Information system (DATASUS), we observed that the number of hospital admissions for PCa increased during the last years until 2019. An average increment of 3.718% for PCa admissions was observed throughout Brazil from 2013 to 2019. However, we observed a significant reduction in the absolute number of hospital admissions for PCa between March 2020 and February 2021 compared to the previous year ( Figure-1 , p <0.0001). There was an 18.7% reduction (6.236 fewer cases) in hospital admissions if compared to the previous year, and a 23.8% reduction (7.916 cases) if considered the projected increment according to the historic increase ( Table-1 ). Hospital admissions for a diagnosis of PCa were also lower during 2020 than in 2019 in all 27 states of Brazil ( Figure-2 ). There was a significant reduction in the number of prostate biopsies performed in 2019, with 11.763 fewer biopsies performed in 2020 (p <0.0001, Table-1 ). This reduction was accompanied by a deficit of over 1.700.000 PSA tests performed in 2020 compared to 2019 (p <0.001) ( Table-1 ; Figure-3 ).

**Table 1:** Effect of the COVID-19 pandemic on Prostate Cancer in Brazil. The impact was the reduction of the diagnosis and procedure involved in this neoplasm compared to previous years and projection.

	2017	2018	2019	2020	Average (2017-2019)	Projection	Déficit* (2019)	Deficit ** (2017-2019)	Deficit *** (projection)	P value
PSA tests	5.193.632	5.495.131	5.751.854	4.020.851	5.480.206	6.053.165	-1.731.003	-1.459.355	-2.032.314	<0.0001
PCa diagnosis	22.396	32.930	39.953	27.358	31.760	53.609	-12.595	-4.402	-26.251	<0.0001
Prostate biopsies	38,23	42.218	41.166	29.403	40.469	42.924	-11.763	-11.066	-13.521	<0.0001
PCa hospital admissions	31,369	33.312	34.680	26.428	33.120	34.344	-8.252	-6.692	-7.916	<0.0001
Radical prostatectomies	8,429	8,829	8.942	6.853	8.733	9.211	-2.089	-1.880	-2.358	<0.0001
Radiotherapy for PCa	-	-	19.824	17.650	19.824	-	-2.174	-2.174	-2.174	0.0079
ADT for PCa	581,544	606,562	636.340	612.725	608.149	665.648	-23.615	4.576	-52.923	<0.0001

*2020 vs. 2019

** 2020 vs. Mean 2017, 2018 and 2019

*** 2020 vs. Historic growth projection

Comparing to the previous year, 2.089 fewer men underwent radical prostatectomies in Brazil (p <0.0001). There was also a significant reduction in the number of men who underwent radiotherapy. There were 2.174 fewer radiotherapies for PCa in 2020 than in 2019. In May 2020, there was an increment of 33.6% in the number of procedures, but in the following months, there was a reduction of up to 15% in the number of procedures compared to 2019. Excluding May, there was a significant reduction of radiotherapies for PCa (p=0.0079). There was also a substantial reduction in ADT for PCa in 2020 compared to previous years, and 23.615 fewer doses of ADT were applied (p <0.0001, Figures-1 ).

We observed a significant reduction in prostate biopsies, hospital admissions, surgical treatments, and radiotherapy treatments for PCa from March 2020 to February 2021 compared to the three previous years in Brazil. There were fewer treatments for localized disease (surgery and radiotherapy) and advanced disease (ADT). The collapse of the Public Health System and social distancing measures may have been associated with this reduction. As previously mentioned, social distancing measures impacted cancer care with the suspension of elective surgeries, procedures, and clinic visits ( 13 ). Several urologic and oncologic associations have proposed guidelines for the treatment of PCa during the COVID pandemic ( 14 - 19 ). In May 2020, the National Comprehensive Cancer Network (NCCN) produced a guideline that encouraged postponing investigation, staging, and treatment of all patients with very low to favorable-intermediate risk PCa. This guideline also recommended that even patients diagnosed with unfavorable intermediate-risk PCa should have their workup deferred until deemed safe. Evidence from a John’s Hopkins retrospective cohort of over 2.300 patients supports this treatment deferral. It showed no association with unfavorable outcomes for patients who have waited for radical prostatectomy for up to six months to treat unfavorable intermediate to very-risk PCa ( 20 ).

There was a significant reduction in Radical Prostatectomies (RP) performed in Brazil, and 2.089 fewer procedures were performed during 2020. (p <0.0001) ( Table-1 ). The American College of Surgeons (ACS), the American Society of Anesthesiologists (ASA), and the American Society of Clinical Oncology have advocated making surgical decisions by a leadership team represented by surgery anesthesiology and nursing departments. Therefore, in April 2020, EAU has produced a list for the triage of urologic surgeries and stated that most RP should indeed be delayed in the face of the aggravation of the pandemic ( 21 ). Following other associations such as NCCN and Canadian Framework, EAU recommended that only surgery for high-risk patients be considered. However, given the availability of other treatments, it was suggested that RP received lower prioritization than other urological surgeries ( 14 , 19 , 21 ). In this scenario, it is possible to understand that the 25% reduction in RP in 2020 when compared to 2019 might be the result of a combination of aspects compounded by the COVID-19 outbreak in Brazil: health system overload with the scarcity of hospital beds; decrease in PCa screening and diagnosis; and lower prioritization of RP in the face of other urological surgeries with redirecting patients to other treatment options. The latter might be implicated in a radiotherapy increase in Brazil’s early stages of the COVID-19 outbreak.

There was an increment during May 2020 compared to May 2019 (from 1.053 to 1.407) in the number of radiotherapies performed for PCa treatment. A possible explanation for the initial increment was the re-management of many patients who were to undergo surgery in the initial phases of the outbreak. But after July 2020, a consistent and significant (p=0.0079) decrease in the number of radiotherapies performed might be an effect of the decline in PCa diagnosis. During the pandemic, the European Association of Urologists (EAU) and NCCN recommended that neoadjuvant androgen-deprivation therapy (ADT) might be considered before external beam radiotherapy (EBRT) for up to 6 months for patients with unfavorable intermediate- to high-risk patients, with 6-month ADT formulations being preferred over 1-month medications ( 18 ). Our data showed a decrease in radiotherapy alongside an increase in adjunctive ADT from May to December 2020 compared to the same period in 2019. This might indicate that patients were preferably sent to neoadjuvant ADT just as recommended by the guidelines during the COVID pandemic.

Of note is the significant reduction of prostate biopsies. In comparison with 2019, there were 11.763 fewer biopsies performed in the year 2020 ( Table-1 , Figure-1 ). Indeed, the Canadian Framework and NCCN recommended that patients with elevated prostatic antigen (PSA) or abnormal digital rectal exam (DRE) might have further testing, and biopsies postponed to the end of pandemic ( 14 , 19 ). Interestingly, our data has shown that a concomitant decrease in the number of PSA tests occurred alongside the reduction in the number of prostate biopsies, especially from March to May 2020, as the COVID-19 outbreak in Brazil deepened its impact ( Figure-3 ).

The reduction in 1.5-2.0 million PSA tests and 11.000-13.000 prostate biopsies during the last year is similar to what happened in the USA after the USPSTF recommendation against PCa screening between 2008-2012 ( 22 ). As a consequence, an increment in metastatic disease was observed during the following years. According to previous studies, for low- and intermediate-risk PCa, a delay in diagnosis and treatment seems to bring little harm in outcomes. However, for high-risk and advanced diseases, treatment delays might have adverse consequences ( 23 ).

Additionally, an economic crisis is currently taking place in Brazil as a consequence of the COVID-19 outbreak. Reduced public expenditure on health care and unemployment are expected. This might result in even higher cancer mortality rates ( 24 ). As a consequence of the significant reduction in diagnostic tests and therapeutic procedures for prostate cancer in Brazil, a cumulative number of patients are expected, and many advanced diseases might be observed during the following years.
